# 

**Published:** 1900-09

**Authors:** 


*The following donations have been received since the publication of the List in June:*

*August 31st, 1900.*

| | |
|---|---|
| The Executor of the late William Adams (1) ... | 6 volumes. |
| The British Medical Association (2) ... ... ... | 113 ,, |
| J. C. Culbertson, M.D. (3) ... ... ... ... ... | 8 ,, |
| L. M. Griffiths (4) ... ... ... ... ... ... ... | 1 volume. |
| Sir William Mac Cormac, Bart. (5) ... ... ... | 1 ,, |
| R. Milne Murray, M.B. (6) ... ... ... ... ... | 1 ,, |
| The Editor of *The Railway Surgeon* (7)... ... ... | 1 ,, |
| E. Noble Smith... ... ... ... ... ... ... ... ... | 1 ,, |
| The Manager of *The Times* (8) ... ... ... ... | 1 ,, |
| The Editor of *Virginia Medical Semi-Monthly* (9) ... | 1 ,, |

A framed picture has been presented by Dr. Robert Roxburgh. Unbound periodicals have been received from Dr. F. H. Edgeworth.

### THIRTY-SEVENTH LIST OF BOOKS.

The titles of books mentioned in previous lists are not repeated.

The figures in brackets refer to the figures after the names of the donors, and show by whom the volumes were presented. The books to which no such figures are attached have either been bought from the Library Fund or received through the *Journal*.

| | | | |
|---|---|---|---|
| **Adams, W.**... ... | *Club-Foot* ... ... ... ... ... ... ... ... | (1) 2nd Ed. | 1873 |
| ,, ... ... | *Lectures on Curvature of the Spine* ... ... | (1) 2nd Ed. | 1882 |
| ,, ... ... | *Surgical Treatment of Deformities* ... ... ... ... | (1) | 1893 |
| **Alt, A.** ... ... | *The Glandular Structures appertaining to the Human Eye* | | 1900 |
| **Auvard, A.**... ... | *100 illustrirte Fälle aus der Frauen-Praxis.* (Fürs Deutsche bearbeitet von A. Rosenau) ... | (2) | 1893 |
| **Bartlett, E.** ... | *On Typhoid and Typhus Fevers* ... ... ... ... | (2) | 1842 |
| **Behrend, H.** ... | *Cattle Tuberculosis and Tuberculous Meat* ... ... | (2) | 1893 |
| **Beissel, J.** [Ed.].. | *Aachen als Kurort* ... ... ... ... ... ... ... ... | (2) | 1889 |
| **Bell, C.** ... ... | *Medico-Legal Studies.* Vol. V. ... ... ... ... | (2) | 1898 |
| **Bell, R.** ... ... | *The Pathogenesis and Treatment of Cancer, without Operation* ... ... ... ... ... ... ... ... ... ... | | 1900 |
| **Böhm und M. von Davidoff, A. A.** | *Lehrbuch der Histologie des Menschen* | (2) | 1895 |

**Braidwood, P. M.** *De la Pyohémie* (Tr. by E. Alling) ... ... ... (2) 1870
**Brodhurst, B. E.** *Anchylosis* ... ... ... ... ... ... ... ... (2) 4th Ed. 1881
**Brunton, T. L.** ... *The Harveian Oration*, 1894 ... ... ... ... ... (2) 1894
**Butlin and W. G. Spencer, H. T.** *Diseases of the Tongue* ... [2nd] Ed. 1900
**Carwardine, T.** ... *Operative and Practical Surgery* ... ... ... ... ... 1900
*Catalogue of the Museum, Royal College of Surgeons, Edinburgh.* Vol. I. (2) 1893
**Chapman, J. A. Wanklyn and E. T.** *Water Analysis*... ... (2) 4th Ed. 1876
**Clark, J.** ... ... *On Consumption and Scrofula* ... ... ... ... ... (2) 1835
**Cock, T. F.**... .. *Manual of Obstetrics*... ... ... ... ... ... ... ... (2) 1853
**Cowen, R. J.** ... *Electricity in Gynæcology* ... ... ... ... ... ... ... 1900
**Dakhyl, H. N.** ... *Du Traitement des Brulures spécialement par l'Acide picrique* ... ... ... ... ... ... ... (2) 2e. Éd. 1898
**Davidoff, A. A. Böhm und M. von** *Lehrbuch der Histologie des Menschen* (2) 1895
**Eade, Sir P.** ... *Notes on Influenza* ... ... ... ... ... ... ... (2) 1891
**Eccles, W. McA.** *Hernia* ... ... ... ... ... ... ... ... ... ... .. 1900
**Fayrer, Sir J.** ... *Recollections of my Life* ... ... ... ... ... ... ... 1900
**Fenwick, E. H.** ... *Ulceration of the Bladder* ... ... ... ... ... ... 1900
**Fothergill, W. E.** *Manual of Midwifery* ... ... ... ... ... 2nd Ed. 1900
*France, Stations hydro-minérales de la* ... ... ... ... ... ... ... ... 1900
**Frankland, P. F.** *Our Secret Friends and Foes* ... ... ... ... ... (2) 1893
**Gairdner, W. T.**... *The Physician as Naturalist* ... ... ... ... ... (2) 1889
**Gerhardt, C.** [Ed.] *Handbuch der Kinderkrankheiten.* Bd. V., Abth. 2 (2) [N.D.]
**Gordon, C. A.** ... *Army Hygiene* ... ... ... ... ... ... ... ... (2) 1866
*Gross, Samuel D., Autobiography of.* Vol. I. ... ... ... ... ... ... (2) 1887
**Hankin, E. H.** ... *The Bubonic Plague* ... ... ... ... ... ... ... (2) 1899
**Hawthorne, C. O.** *Rheumatism, Rheumatoid Arthritis, and Subcutaneous Nodules* ... ... ... ... ... ... ... ... ... ... 1900
**Hertoghe, E.** ... *De l'Hypothyroïdie bénigne chronique ou Myxœdème fruste* ... ... ... ... ... ... ... ... (2) 1899
**Holmes, T.** ... ... *Introductory Address delivered at St. George's Hospital, October 2nd,* 1893, *on the Centenary of John Hunter's Death* ... ... ... ... ... ... ... ... (2) 1893
**Horton-Smith, P.** *The Typhoid Bacillus and Typhoid Fever* ... ... ... 1900
**Huber, J. C.** ... *Bibliographie der klinischen Helminthologie* (2) Hefts V.—IX. (in 3) 1893-95
**Jones, H. L.** ... *Medical Electricity* ... ... ... ... ... ... 3rd Ed. 1900
**Locke, J.** ... ... *An Essay concerning Human Understanding* ... ... (2) 1828
**Lowndes, W. T.**... *The Bibliographer's Manual* ... ... 6 vols. (in 11) 1857-64
**MacCormac, Sir W.** *Address of Welcome—Centenary of the Royal College of Surgeons of England*... ... ... ... ... ... (5) 1900
**Macewen, W.** ... *Die Infectiös-Eiterigen Erkrankungen des Gehirns und Rückenmarks* (Deutsche ausgabe von P. Rudloff) (2) 1898
**Manson, P.**... ... *Tropical Diseases* ... ... ... ... ... [2nd] Ed. 1900
**Marcet, W.** ... *Diseases of the Larynx* ... ... ... ... ... ... (2) 1869
**Mitchell, C. A.** ... *Flesh Foods* ... ... ... ... ... ... ... ... ... ... 1900
**Newman, G.** ... *Bacteria* ... ... ... ... ... ... ... ... ... ... ... 1899
**Nicolaier, A.** ... *Urotropine*... ... ... ... ... ... ... ... ... ... (4) [N.D.]
*Paris et l'Exposition, Renseignements pratiques sur* ... ... ... ... ... 1900
*Paris-Médical* ... ... ... ... ... ... ... ... ... ... ... ... ... ... 1900

**Park, W. H.** ... *Bacteriology in Medicine and Surgery* ... ... ... ... ... 1900
**Parkin, J.** ... ... *Epidemiology.* Part I. ... ... ... ... ... ... (2) 1873
*Pathology, An Atlas of Illustrations of* (New Syd. Soc.) Fasc. XIII. ... 1900
*Pharmacopœia, Prescribers'* ... ... ... ... ... ... ... ... ... (2) 3rd Ed. 1896
**Poland, J.** ... ... *Records of the Miller Hospital and Royal Kent Dispensary*... ... ... ... ... ... ... ... ... (2) 1893
"*Punch,*" *An Evening with* .. ... ... ... ... ... ... ... ... (8) 1900
**Schaeffer, O.** ... *Atlas der Geburtschilfe.* Theil II.... ... ... ... (2) 1892
*Selected Essays and Monographs* (New Syd. Soc.) ... ... ... ... ... ... 1900
**Sibthorpe, C.** ... *Clinical Manual for India* ... ... ... (2) 3rd Ed. 1890
**Sieveking, E. H.** *The Medical Adviser in Life Assurance*... (2) 2nd Ed. 1882
**Spencer, D. B.** ... *A Record of Indian Fevers* ... ... ... ... ... (2) 1899
**Spencer, H. T. Butlin and W. G.** *Diseases of the Tongue* ... [2nd] Ed. 1900
**Squire, P.** ... ... *The Pharmacopœias of Thirty of the London Hospitals* (Ed. by P. W. Squire) ... ... ... ... 7th Ed. 1900
**Steven, J. L.** ... *Clinical Medicine* ... ... ... ... ... ... ... ... 1900
**Swan, J.** ... ... *Diseases and Injuries of the Nerves* ...(2) [2nd Ed.] 1834
,, *On the Means employed for correcting the Inverted Image on the Retina of the Eye* ... ... ... ... ... (2) 1865
**Swanzy, H. R.** ... *Diseases of the Eye* ... ... ... ... ... ... 7th Ed. 1900
**Thomson, A.** ... *On Neuroma and Neuro-Fibromatosis* ... ... ... ... 1900
**Virchow, R.** [Ed.] *Handbuch der speciellen Pathologie und Therapie* Bd. II. ... ... ... ... ... ... ... ... ... (2) 1855
**Walsham, W. J.** *Surgery: its Theory and Practice* ... ... ... 7th Ed. 1900
**Wanklyn and E. T. Chapman, J. A.** *Water Analysis* ... ... (2) 4th Ed. 1876
**Whistler, W. Mac N.** *Syphilis of the Larynx* ... ... ... ... ... (2) 1879
**Zerolo, T.** ... ... *Climatoterapia de la Tuberculosis pulmonar* ... ... (2) 1889

### TRANSACTIONS, REPORTS, JOURNALS, &c.

American Gynæcological and Obstetrical Journal, The Vols. XV., XVI. 1899-1900
American Gynecological Society, Transactions of the Vols. XIII.-XV., 1888-90, XVII.-XIX. 1892-94
American Journal of Obstetrics, The ... ... ... ... ... Vol. XXIV. 1891
American Journal of Ophthalmology, The ... ... ... ... Vol. XVI. 1899
American Journal of the Medical Sciences, The ... N.S., Vol. CXIX. 1900
American Laryngological Association, Transactions of the, for 1893 (2) 1894
American Medico-Psychological Association, Proceedings of the ... (2) 1898
American Orthopedic Association, Transactions of the (2) Vols. X., XI. 1897-98
American Practitioner and News, The ... ... Vols. XXVII., XXVIII. 1899
American Year-Book of Medicine and Surgery, The ... ... 2 parts 1900
Annales de la Société Belge de Chirurgie ... ... ... ... Tome VII. 1899
Annales de l'Institut de Pathologie et de Bactériologie de Bucarest. Vol. VI. 1898

Annali di Ostetricia e Ginecologia ... ... ... ... ... ... ... ... 1899
Annual and Analytical Cyclopædia of Practical Medicine ... Vol. V. 1899
Archiv für Verdauungs-Krankheiten ... ... ... ... ... ... Bd. V. 1899
Archives of Ophthalmology ... Vols. VIII., IX., 1879–80, XI.–XIII. 1882–84
Archives of Ophthalmology and Otology ... ... ... ... ... Vol. V. 1876
Archives of Otology ... ... ... Vols. VIII., IX., 1879–80, XI.–XIII. 1882–84
Archives of Surgery ... ... ... ... ... ... ... ... ... ... Vol. X. 1899
Archives provinciales de Chirurgie ... ... ... ... ... Tome VIII. 1899
Archives provinciales de Médecine ... ... ... ... . . ... Tome I. 1899
Archivio di Ortopedia ... ... ... ... ... ... ... ... [Vol. XVI.] 1899
Army Medical Department—Reports ... ... ... (2) Vols. VI., 1866,
XXV.–XXVII., 1885–87, XXIX., 1889, XXXII., 1892, XXXIV. 1894
Association of American Physicians, Transactions of the (2) Vol. IX. 1894
Australasian Medical Gazette, The ... ... ... ... ... Vol. XVIII. 1899
Bombay, Report on the Bubonic Plague in, 1896–97 .. ... ... (2) 1897
" " " Plans, Temperature
Charts, &c. ... ... ... ... ... ... ... .. ... 2 vols. (2) 1897
Boston Medical and Surgical Journal, The ... ... ... ... Vol. CXLII. 1900
British Gynæcological Journal, The ... ... ... ... ... ...Vol. XV. 1899
British Medical Journal, The ... ... ... ... ... ... ... Vol. I. for 1900
Bulletin de l'Académie de Médecine ... 3e Sér., Tomes XLI., XLII. 1899
Bulletin de l'Académie royale de Médecine de Belgique 4e Sér.,
Tome XIII. 1899
Canadian Practitioner and Review, The ... ... ... ... Vol. XXIV. 1899
Centralblatt für innere Medicin ... ... ... ... ... ... ... ... ... 1899
Cincinnati Lancet and Observer, The (3) N.S., Vols. II., 1859,
VI., 1863, VIII., 1865, XIV., 1871, XVIII.–XXI. 1875–78
Clinical Journal, The ... ... ... ... ... ... ... ... Vol. XV. 1900
Columbia University, Studies from the Department of Pathology of
the College of Physicians and Surgeons (2) Vol. V., Part 1. 1896-97
Diphtheria, Report on—School Board for London ... ... ... ... (2) 1896
Edinburgh Obstetrical Society, The Transactions of the Vols. IV.,
1878; (6) V., Part 1, 1878; VI.–X. 1881–85
Epidemiological Society of London, Transactions of the
(2) Vol. IV., Part 4. 1882
France médicale, La ... ... ... ... ... ... ... ... ... ... ... ... ... 1899
Gazette médicale de Paris ... ... ... ... ... 11e Sér., Tome II. 1899
Gazzetta médica Lombarda ... ... ... ... ... ... ... ... ... ... 1899
Glasgow Medical Journal, The ... ... ... ... ... ... Vol. LIII. 1900
Grèce médicale, La ... ... ... ... ... ... ... ... ... ... ... ... ... 1899
Hospital, The ... ... ... ... ... ... ... ... ... ... Vol. XXVII. 1900
Hospitals—
Middlesex Hospital, The—Registrars' Reports for (2) 1871,
1875, 1881, 1887
Montreal General Hospital Reports ... ... ... ... (2) Vol. I. 1880

Hospitals and Charities, Burdett's ... ... ... ... ... ... ... ... ... ... 1900

Hydrophobia — Report of a Committee appointed by the Local Government Board on Pasteur's Treatment ... ... ... ... (2) [1887]

India, Scientific Memoirs by Medical Officers of the Army of (2) Part II. 1898

Indian Medical Gazette, The ... ... ... ... ... ... ... ... Vol. XXXIV. 1899

Intercolonial Medical Journal of Australasia ... ... ... . Vol. IV. 1899

International Medical Magazine ... ... ... ... ... ... ... Vol. VIII. 1899

Johns Hopkins Hospital Bulletin, The ... ... ... ... ... ... ... Vol. X. 1899

Journal of Balneology and Climatology, The ... ... ... ... Vol. III. 1899

Journal of Public Health ... ... ... ... ... ... ... (2) Vols. I., II. 1855–56

Journal of the Sanitary Institute ... ... ... ... ... ... Vol. XX. 1900

Kentucky State Medical Society, Transactions of the N.S., Vol. VIII. 1900

Laboratory Reports, Royal College of Physicians, Edinburgh Vol. VII. 1900

Lancet, The ... ... ... ... ... ... ... ... ... ... ... Vol. I. for 1900

Laryngoscope, The ... ... ... ... ... ... ... ... Vols. VI., VII. 1899

Library Association Year Book, The .. ... ... ... ... ... ... ... 1900

Local Government Board—Reports on the Port and Riparian Sanitary Survey of England and Wales, 1893–94 ... ... ... ... ... ... (2) 1895

London County Council—Annual Reports of the Medical Officer of Health ... ... ... ... ... (2) 1895, 1897, 1898

,, ,, ,, —Report on the Treatment of Sewage ... (2) [N.D.]

Marine Hospital Service of the United States, Annual Report of the Supervising Surgeon-General of the, for 1895 and 1896 2 vols. (2) 1896

Medical Age, The ... ... ... ... ... ... ... ... ... ... Vol. XVII. 1899

Medical Libraries ... ... ... ... ... ... ... ... ... ... Vols. I., II. 1899

Medical News, The ... ... ... ... ... ... ... ... ... ... Vol. LXXVI. 1900

Medical Press and Circular, The ... ... ... ... ... ... ... [Vol. CXX.] 1900

Medical Record ... ... ... ... ... ... ... ... ... ... ... Vol. LVII. 1900

Mercy and Truth ... ... ... ... ... ... ... ... ... ... ... Vol. III. 1899

Metropolitan Asylums Board—Reports of Statistical Committees, &c., for (2) 1893, 1894; (2) 1898, 2 vols., 1899

Monatshefte für praktische Dermatologie ... ... ... (2) Bd. XVII. 1893

Montreal Medical Journal, The ... ... ... ... ... ... Vol. XXVIII. 1899

Monthly Cyclopædia of Practical Medicine, The ... ... ... Vol. II. 1899

Monthly Gazette of Health, The ... ... ... (2) Vols. I.–IV. (in 2) 1817–19

New York Medical Journal, The ... ... ... ... ... ... ... Vol. LXXI. 1900

Northumberland and Durham Medical Journal, The ... ... ... ... 1899

Occidental Medical Times ... ... ... ... ... ... ... ... Vol. XIII. 1899

Pan-American Medical Congress, Transactions of the First (2) 3 parts 1895

Philadelphia County Medical Society, Proceedings of the Vol. XX. 1899

Post-Graduate, The ... ... ... ... ... ... ... ... ... ... Vol. XIV. 1899

Practitioner, The ... ... ... ... ... ... ... ... ... ... Vol. LXIV. 1900

Progressive Medicine ... ... ... ... ... ... ... ... ... Vols. I., II. 1900

| | | |
|---|---|---|
| Quarterly Journal of Foreign Medicine and Surgery, The | (2) Vol. I. | 1819 |
| Railway Surgeon, The ... ... ... ... ... ... ... ... ... | (7) Vol. VI. | 1900 |
| Receuil des Travaux du Comité consultatif d'Hygiène publique de France | (2) Tome XXVII. | 1898 |
| Retrospect of Medicine, Braithwaite's ... ... ... .. ... | Vol. CXXI. | 1900 |
| Revue générale d'Ophtalmologie ... ... ... ... ... | Tome XVIII. | 1899 |
| Revue hebdomadaire de Laryngologie, d' Otologie, et de Rhinologie | Tome XX., Vol. 1. | 1900 |
| St. Petersburger medicinische Wochenschrift ... ... ... ... ... ... ... | | 1899 |
| Sanitary Commission, First Report of the Royal ... ... ... ... | (2) | 1869 |
| Sanitary Record, The | Vols. VIII., 1887, X.—XIII., 1889-92, XXV. | 1900 |
| Sanitary Review, The ... ... ... ... ... ... | (2) Vols. III., IV. | 1857-58 |
| Scottish Medical and Surgical Journal, The ... ... ... ... | Vol. VI. | 1900 |
| Sei-I-Kwai Medical Journal, The ... ... ... ... ... ... ... | Vol. XVIII. | 1899 |
| Semaine médicale, La ... ... ... ... ... ... ... ... ... ... ... | (2) | 1891 |
| Therapeutic Gazette, The ... ... ... ... ... ... ... ... | Vol. XXIII. | 1899 |
| Vaccination, Papers on—General Board of Health ... ... ... ... | (2) | 1857 |
| Virginia Medical Semi-Monthly ... ... ... ... ... ... | (9) Vol. IV. | 1900 |
| West Riding Lunatic Asylum, The—Medical Reports | Vols. I., II., 1871-72, VI. | 1876 |
| Year-Book of Scientific and Learned Societies ... ... ... ... ... .. | | 1900 |
| Zeitschrift für Krankenpflege ... ... ... ... ... ... ... ... ... | | 1899 |

### Local Medical Notes.

### THE PRESENTATION TO MR. L. M. GRIFFITHS.

As many of the subscribers to the testimonial to Mr. L. M. Griffiths may like to know something of the details of the bookplate which formed part of the presentation, we give a reproduction of it [1] and some remarks on it supplied by the artist, Mr. W. V. Collette.

Of late years the armorial bookplate has to a great extent been superseded, and it is now the custom to show, in the place of arms, crest, or heraldic blazonry, a design that tells something of the profession, pursuits, and tastes of the owner. In the majority of cases this is a comparatively easy task for the designer, but when the owner is a man of many parts it becomes a matter of considerable difficulty to introduce in the very limited space at the artist's disposal the various objects which symbolise the subjects in which his client is specially interested.

1 The lettering on the volumes can be distinctly read in the impressions taken from the copper, but in the reproduction some of the words have become blurred.

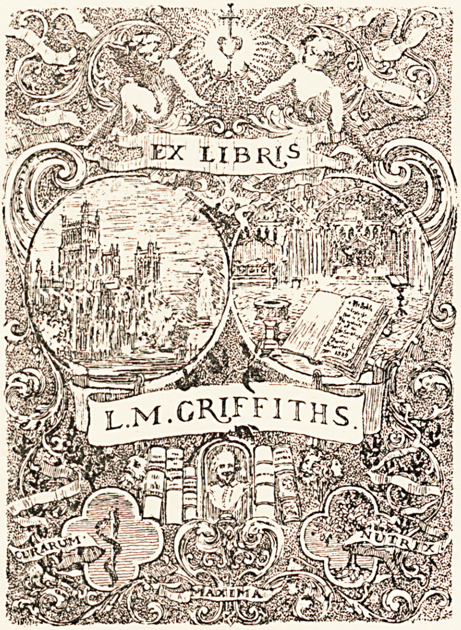

EX LIBRIS

L.M. GRIFFITHS.

CURARUM

NUTRIX

MAXIMA

In this instance the profession of the owner is represented by the Æsculapian rod and serpent, and by the tree of life in the quatrefoils in the lower portion of the design. The introduction of Tanner's *Practice of Medicine*, a book much in vogue some years since, and of Heath's *Minor Surgery* shows that the possessor of the bookplate is a general practitioner in whose work surgery has only a subordinate place. The view of Bristol Cathedral indicates not only the city in which he lives, but also his churchmanship, and this is further emphasised by a representation of some of the more sacred things to be found in the interior. The statue of Queen Victoria, which was erected near the Cathedral, has also been included, and serves to point out the loyalty of the owner of the bookplate to the form of government under which this country has flourished so well.

Opportunity has been taken to give in addition to the open Bible on the lectern a portion of the title-page of the Great Bible of 1539, not only because this was the chief precursor of the various Bibles in English which appeared about that time, but also because the library of which this is to be the bookplate contains a great deal of literature on the Bible. The choice of this epoch-making book shows that the attention of the owner of the bookplate is not chiefly directed to the languages in which the Book was originally written, but to our own version, and this is still further pointed out by the introduction of the volume containing the parallel texts of the Authorised and Revised Versions of the New Testament.

Some of the other characteristics of the owner's library are indicated by Hook's *Church Dictionary* and by the Works of Shakspere, a reproduction of whose bust from the monument in the Church of Stratford-on-Avon is also given. The possession of books referring to the Drama generally is pointed out by the masks of Comedy and Tragedy, and the mask of History is added to represent one branch of dramatic literature and to still further indicate the nature of the library.

The motto, "Curarum maxima Nutrix," is appropriated from Ovid's description of Night (*Metam.*, viii. 81), and is particularly applicable to the books which are a delight and a refreshment to their owner, and which, as a restorative, rival that which "knits up the ravell'd sleave of care" and is "chief nourisher in life's feast."

The emblematic design of the conjoined cross, anchor, and heart—behind which is the sun, representing light and knowledge—is introduced as indicating that which should dominate the life of all of us, while the recumbent effigy in the canopied tomb in the circle on the right is a useful *memento mori*.

Brilliancy is given to the whole design by introducing here and there in its background minute bay-leaves of a deeper tone than the other portions of the plate, and its general artistic effect is heightened by the cherubic figures introduced into its upper portion.

---

### BRISTOL.

University College, Bristol.—The Associateship of the College has been conferred upon Mr. M. Bergin, M.B. Lond., and Mr. A. R. Short, B.Sc. Lond.

Mr. C. O. Bodman, M.R.C.S., L.R.C.P., recently took the degrees of M.B. and B.S. of the University of Durham, and the following gentlemen have obtained the diplomas of the M.R.C.S. Eng., and L.R.C.P. Lond., viz.:—Messrs. L. A. Moore, J. M. H. Munro, D.Sc., M. H. Phillips, W. B. Prowse, C. F. Walters, and S. R. Williams.

At the Intermediate Examination in Medicine of the London University, held in July, Messrs. Henry Devine and A. R. Short were successful. Mr. J. H. Parsons, M.B. Lond., M.R.C.S., L.R.C.P., has passed the Primary Examination of the F.R.C.S. Eng. Diploma.

Mr. Arthur Rendle Short, by obtaining the first place in Physiology and Histology with the Exhibition and Gold Medal, the first place in Materia Medica with the Exhibition and Gold Medal, and first class honours in Anatomy, has done great credit to himself and to the College to which he belongs.

A Clinical and Bacteriological Research Laboratory has been established at the College with the object of "enabling the medical men of Bristol and the West of England to obtain reliable information and reports upon pathological material, and of placing at the disposal of authorities dealing with drinking water, persons concerned with the supply or consumption of milk, and those engaged in manufacturing processes, the resources of a properly equipped Research Laboratory. Medical men and others will be afforded the opportunity of personally carrying out investigations, should they desire it."

It will be noticed that work is undertaken for medical men, and also for others not members of the profession. For the former the ordinary investigations of a Clinical Laboratory will be carried on, such as the examination of Sputum, Urine, Pus, &c., as well as of Serum for the Widal reaction, and of throat secretions for Diphtheria Bacilli; and facilities will be given to those who wish to carry out work on their own account. For those not members of the profession the work will vary with the requirements, and it is impossible to enumerate every case in which the Laboratory may be of service; but whenever doubt arises it is suggested that inquiries be made, when an opinion will be given as to whether good is likely to come of any investigation which can be undertaken.

This Laboratory has been organised by, and will be under the direct supervision of, Professor A. F. Stanley Kent, Professor of Physiology at the College and Bacteriologist to the Royal Infirmary.

Bristol Royal Infirmary.—We have observed with much regret the announcement of Mr. F. Richardson Cross's resignation of the office of ophthalmic surgeon at the Bristol Royal Infirmary, which he has held for fifteen years, previous to which he filled the posts of assistant surgeon and surgeon for a period of seven years. His energy and enthusiasm in all matters relating to the interests of his profession, and his valuable teaching, will still be available to his *confrères* in Bristol, where he will continue to carry on his work, although the increasing pressure of engagements has caused him to sever his connection with the active work of the Royal Infirmary, to which he has been elected Honorary Consulting Ophthalmic Surgeon.

Princess Christian Hospital.—We note with pleasure the safe return of various members of the staff whose departure was announced in a former issue. Mr. Paul Bush, Mr. Flemming, and Mr. Cridland have resumed their duties in Bristol, but Mr. Pearse remains as one of the staff of the Princetown Hospital in Natal.

The 2nd Gloucester Royal Engineer Volunteer Corps.—The new company formed entirely by students and members of the staff of University College, Bristol, is now nearly up to full strength. Professor A. F. Stanley Kent has been commissioned First Lieutenant of the company.

Cyclopia.—This successful festival was held at the Clifton Zoological Gardens on June 28th, 29th, and 30th, with the object of obtaining funds for the extensions which have lately been made at the Bristol Eye Hospital. We are pleased to hear that the substantial sum of about £1,260 will be contributed to the required funds as the result of the undertaking. The extensions which have been made at the Hospital have involved the adding of an entirely new storey to the building, providing proper sleeping accommodation for the nurses and servants, some small wards for patients, and a thoroughly modern operating theatre. Also a new wing has been built at the back, and the whole Hospital fitted with the electric light. The total cost of the extensions will exceed £6,000. They will be opened at the end of September. The numerous attractions for the public afforded by the Cyclopia assured success, and if the weather had not greatly interfered with the proceedings on the third day this success would have been even much greater.

Public Health.—The Health Committee of Bristol propose to submit to the Town Council a scheme for increasing the accommodation for infectious diseases. At present 147 beds are available, which is an average of one bed for 2,300 of the population. The Health Committee suggest the extension of Ham Green Fever Hospital by the addition of the buildings originally intended, and in the meantime temporarily to re-open Clift House, Bedminster, as a hospital, in case the present hospital should not afford the necessary accommodation.

### BARNSTAPLE.

North Devon Infirmary.—The annual meeting of the subscribers and friends of the North Devon Infirmary was held on August 21st, under the presidency of Lord Fortescue. The report stated that during the past twelve months 654 in-patients and 2,269 out-patients had been treated. The average daily number of in-patients was 46, the average stay of each being 25½ days. The financial statement showed that the total ordinary expenditure exceeded the ordinary income by about £500. The committee reported that the Convalescent Home in connection with the Infirmary at Morthoe was doing a most useful work.

### BARRY.

Small-pox Hospital.—At a meeting of the Public Health Committee of the Barry District Council, held on August 6th, it was decided to erect a permanent small-pox hospital at an estimated cost of £8,160.

### CARMARTHEN.

Obituary.—Mr. William Lewis Hughes died at his residence, Carmarthen, on July 17th, in his 48th year. He received his medical education at the London Hospital Medical School, qualifying as

M.R.C.S. Eng. and L.S.A. Amongst other local appointments, Mr. Hughes was consulting surgeon to the Carmarthen Infirmary and medical officer of health of the town.

### MERTHYR TYDVIL.

Workhouse Infirmary.—The new Workhouse Infirmary is now completed, and is occupied by the patients. The female wards contain 42 beds, and there is also a lying-in ward of 10 beds. The men's wards provide accommodation for 70 beds. The expenditure has been over £13,000, exclusive of the furnishing, which amounted to £1,300.

### SWANSEA.

General Hospital.—The annual general meeting of this institution was recently held under the presidency of Colonel Morgan. The report stated that 892 in-patients had been admitted during 1899, the daily average number of occupied beds being 90.1, against 84 in 1898. The out-patients numbered 4,392. The financial statement showed that during the year the adverse balance was reduced from £1,391 to £845. The annual income was greater by £244. The workpeople's contributions showed an increase of £359. Baroness Patti-Cederstrom's concert realised £360 for the charity, and the house-to-house collections reached £418. During the year several much-needed improvements have been carried out in the building.

### SWINDON.

Lunacy.—At the meeting of the Board of Guardians, held on August 2nd, it was reported that 142 patients from this union were inmates of the Wiltshire County Asylum, the cost for their maintenance being £3,400.

### TEIGNMOUTH.

Hospital.—Mr. T. E. Frazer-Toovey has been appointed House-Surgeon.

### TRURO.

Royal Cornwall Infirmary.—The annual meeting of this institution was held on August 13th, under the presidency of Colonel Tremayne. The report showed that during the past year 393 in-patients had been admitted; 790 out-patients had also been attended, against 650 in the previous year. The financial statement showed that the receipts amounted to £1,902, and there was a favourable balance of £86 remaining. The Cornwall Convalescent Home for Men at Perranporth, in connection with the Infirmary, had received 114 patients, their average stay being 24 days, and the cost per patient £2 5s. 8d. The High Sheriff of Cornwall (Mr. R. Harvey) was elected president for the ensuing twelve months.

